# Supplementary material for: Therapeutic Peptide RF16 Derived from CXCL8 Inhibits MDA-MB-231 Cell Invasion and Metastasis
Source: Int J Mol Sci. 2023 Sep 13;24(18):14029. doi: 10.3390/ijms241814029 (PMC10531501; doi:10.3390/ijms241814029)
Supplement: Supplementary file 1 [file ijms-24-14029-s001.zip › ijms-2587721-supplementary.pdf]

A

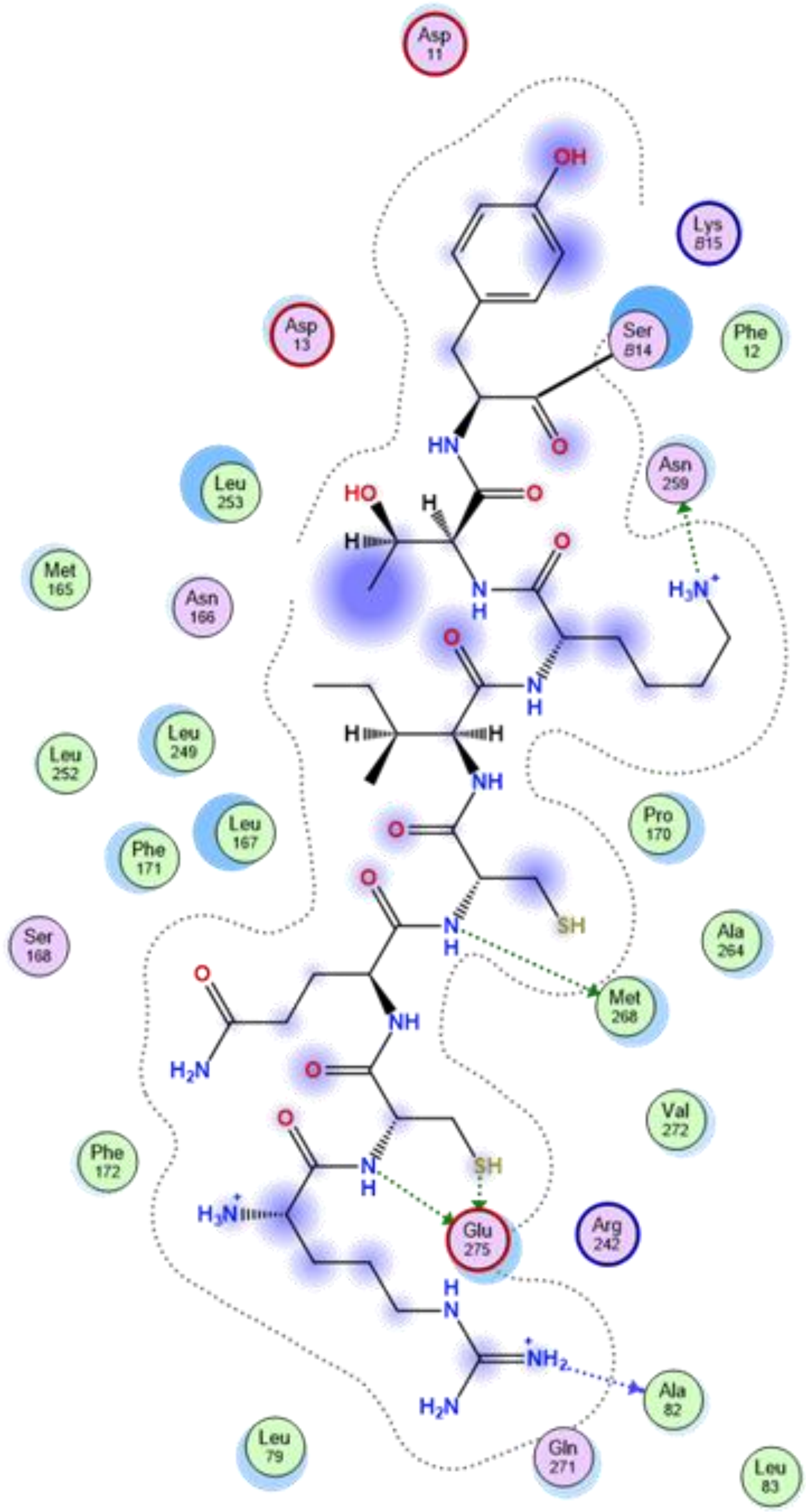

Asp 11
OH
Lys B15
Asp 13
Ser B14
Phe 12
HN
O
Asn 259
Leu 253
HO
H
O
H
O
Met 165
Asn 166
N
H
H_3N^+
O
N
H
H
H
Leu 249
Leu 252
NH
Pro 170
Leu 167
O
Phe 171
O
SH
Ser 168
N
H
Ala 264
O
NH
Met 268
H_2N
O
Val 272
O
Phe 172
N
H
SH
H_3N^+
Glu 275
Arg 242
H
N
NH_2^+
Ala 82
H_2N
Leu 79
Gln 271
Leu 83

B

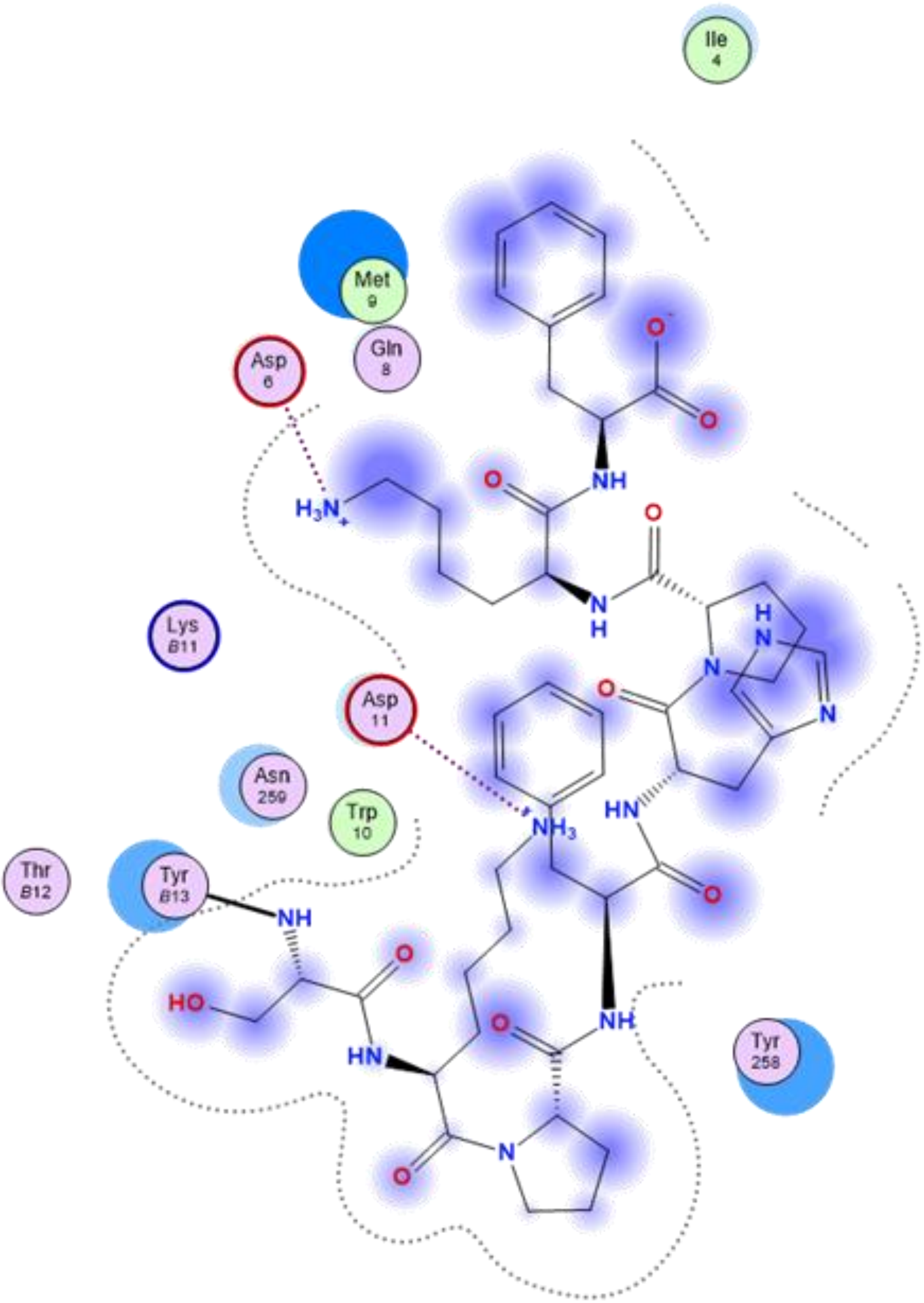

Ile
4
Met
9
Gln
8
Asp
6
Lys
B11
Asp
11
Asn
259
Trp
10
Thr
B12
Tyr
B13
Tyr
258

C

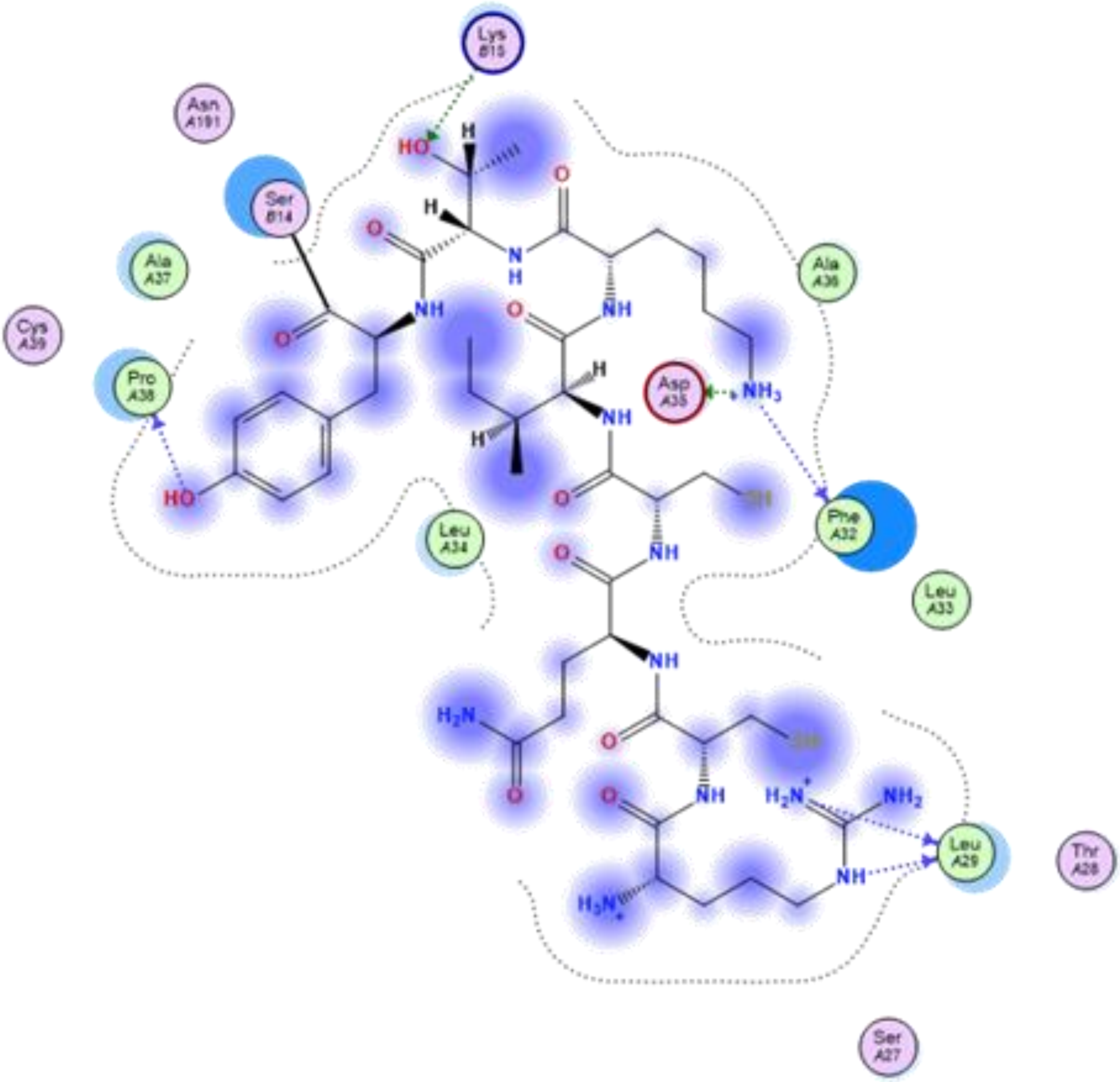

Lys B15
Asn A191
Ser B14
Ala A37
Cys A39
Pro A38
Ala A36
Asp A35
Phe A32
Leu A34
Leu A33
Leu A29
Thr A28
Ser A27

D

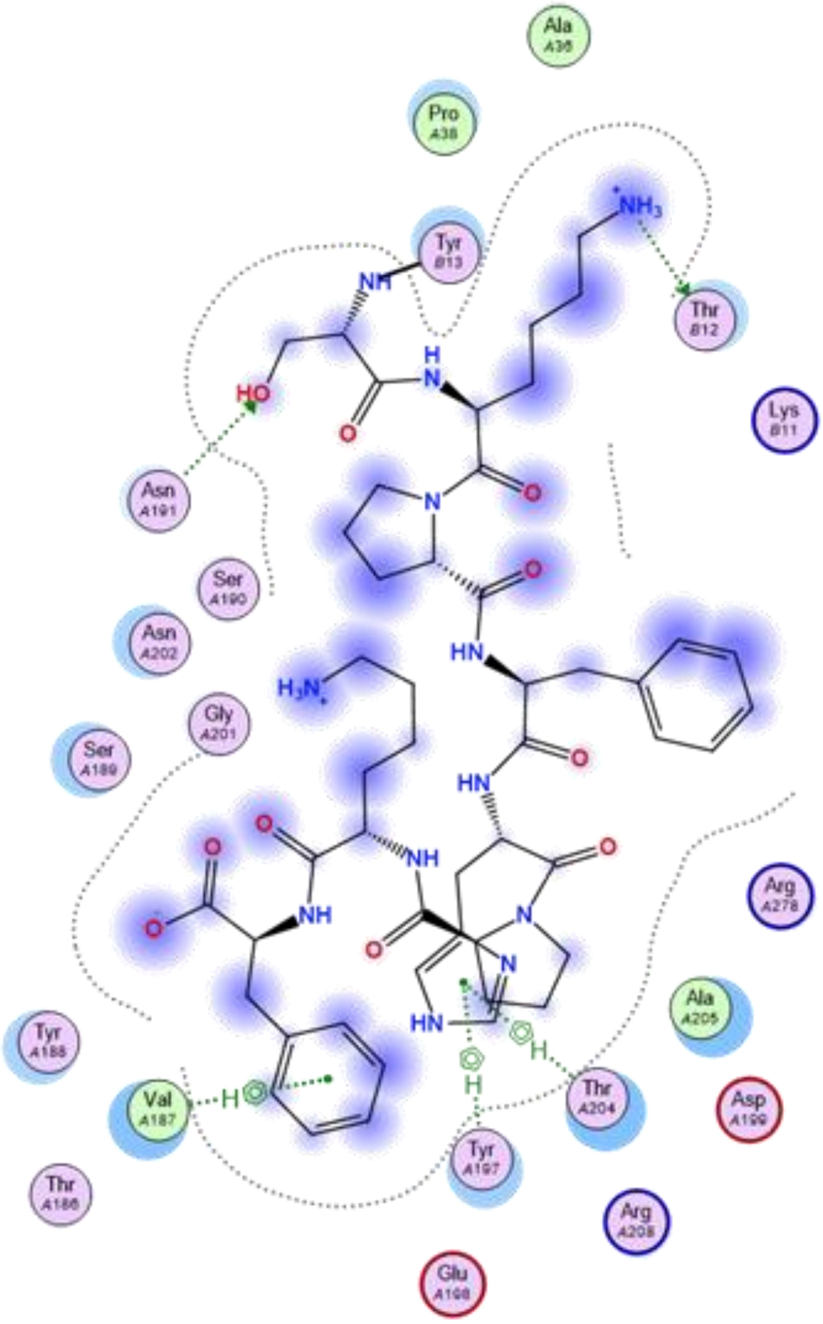

Supplementary Figure S1. 2D position analysis of Figure 1. The RF-16 peptide sequence is RCQCIKTYSKPFHPKF. Here it shows is the map of ligand to receptor interaction. the result is divided into 4 parts:

A. N-terminal (bottom; R to Y) binding to CXCR1

B. C-terminal (top; S to F) binding to CXCR1

C. N-terminal (bottom; R to Y) binding to CXCR2

D. C-terminal (top; S to F) binding to CXCR2
